# Blockers of Skeletal Muscle Na_v_1.4 Channels: From Therapy of Myotonic Syndrome to Molecular Determinants of Pharmacological Action and Back

**DOI:** 10.3390/ijms24010857

**Published:** 2023-01-03

**Authors:** Michela De Bellis, Brigida Boccanegra, Alessandro Giovanni Cerchiara, Paola Imbrici, Annamaria De Luca

**Affiliations:** Section of Pharmacology, Department of Pharmacy-Drug Sciences, University of Bari “Aldo Moro”, 70125 Bari, Italy

**Keywords:** sodium channel, skeletal muscle, myotonia, mexiletine, SAR

## Abstract

The voltage-gated sodium channels represent an important target for drug discovery since a large number of physiological processes are regulated by these channels. In several excitability disorders, including epilepsy, cardiac arrhythmias, chronic pain, and non-dystrophic myotonia, blockers of voltage-gated sodium channels are clinically used. Myotonia is a skeletal muscle condition characterized by the over-excitability of the sarcolemma, resulting in delayed relaxation after contraction and muscle stiffness. The therapeutic management of this disorder relies on mexiletine and other sodium channel blockers, which are not selective for the Na_v_1.4 skeletal muscle sodium channel isoform. Hence, the importance of deepening the knowledge of molecular requirements for developing more potent and use-dependent drugs acting on Na_v_1.4. Here, we review the available treatment options for non-dystrophic myotonia and the structure–activity relationship studies performed in our laboratory with a focus on new compounds with potential antimyotonic activity.

## 1. Introduction

The voltage-gated sodium channels (VGSCs) (Nav1.1-Nav1.9 family) represent a valuable source for further drug development for the treatment of many diseases in which the Nav channel may be directly or indirectly involved [1]. In excitable cells like neurons, cardiac and skeletal muscle cells, the concerted activity of specific isoforms of Nav channels, Na^+^/K^+^-ATPase, and sodium exchangers contribute to maintaining sodium homeostasis and play a critical role in some physiological activities such as action potential generation, muscle contraction, neurotransmitter, and hormone secretion and cognitive processes [2]. The expression of multiple Nav channel subtypes in non-excitable tissues such as immune cells of the myeloid lineage, glial cells, fibroblasts [3,4], T lymphocytes, osteoblasts, and endothelial cells shed light on other underexplored functions. For example, Nav channels are thought to affect endosomal acidification in phagocytic cells and podosome formation in migratory immune cells [3].

To date, more than 1000 point mutations have been detected in the Nav channels isoforms expressed in the central and peripheral nervous systems, heart, and skeletal muscle, thus linking Nav channel genes to a wide spectrum of diseases comprising epilepsy, migraine, pain, myotonia and paralysis, diabetes, autism, cardiovascular diseases [5,6,7,8]. Intriguingly, an abnormal expression of Nav channels has been described in cancer cells from, for example, breast, colon, prostate, and non-small cells lung tumors; although this alteration can be part of the complex genetic background of cancer cells, the possible involvement of Na channels in tumor growth, invasion and metastasis is an active research area to identify novel biomarkers and drug targets [9,10,11,12].

Na_v_1.4, encoded by the *SCN4A* gene, is the specific skeletal muscle sodium channel isoform. Na_v_1.4 channel expression increases during muscle development and cell differentiation, corroborating its key role in priming myofiber phenotypes in response to neuronal input [13]. In fact, during denervation, rat myofibres have increased permeability of sodium [14] in part related to a transient re-expression of the juvenile/cardiac sodium channel Nav1.5, which declines afterward [15]. In addition, Na_v_1.4 channel expression and function increase during myoblast to myotube formation, suggesting that they could also be implicated in the myogenic process [16].

Mutations in Na_v_1.4 have been associated with various neuromuscular disorders, including *SCN4A*-related myotonia, paramyotonia congenita of von Eulenburg, congenital myasthenic syndrome, hypokalemic periodic paralysis type 2, hyperkalemic periodic paralysis and normokalemic periodic paralysis [17,18]. Muscle excitability may be either pathologically enhanced or reduced as a consequence of Na_v_1.4 gain-of-function mutations leading to non-dystrophic myotonias (NDMs) or periodic paralysis, respectively. Distinct from myotonic dystrophy, NDMs are skeletal muscle disorders characterized by skeletal muscle firing a burst of action potentials, delayed relaxation after contraction and muscle stiffness. Conversely, periodic paralysis is characterized by muscle weakness. Impaired fast inactivation of Na_v_1.4 mutants likely induces myotonia, while enhancement of activation and impaired slow inactivation contribute to paralytic attacks [19,20]. Loss-of-function mutations in the skeletal muscle chloride channel ClC-1 (encoded by the *CLCN1* gene), cause another form of hereditary myotonia, namely myotonia congenita. The role played by this ion channel in skeletal muscle electrical activity is opposite to that of Na_v_1.4: while Na_v_1.4 current renders the membrane excitable, allowing it to fire action potentials, ClC-1 current dampens excitability and stabilizes the resting membrane potential [21,22]. Aberrant sodium currents have also been reported in skeletal muscle and cardiac disorders, likely as a secondary defect consequent to a different primary cause (Duchenne muscular dystrophy and angina pectoris, heart failure) [23].

Before discussing the structure-based development of drugs acting on the Na_v_1.4 channel, it is worth mentioning the therapeutic options for non-dystrophic myotonias and the structural basis for sodium channel block by small molecules.

## 2. Sodium Channel Blockers as Therapeutic Options in Non-Dystrophic Myotonias

The current therapeutic approach to treat non dystrophic myotonias, regardless of the underlying channel defect, be it ClC-1 or Na_v_1.4, is based on available sodium channel blockers that work by lowering sarcolemmal excitability. In fact, whereas no drug able to increase ClC-1 activity is currently available, several sodium channel blockers are approved, including anti-arrhythmic drugs, antiepileptics and local anesthetics (LAs), some used off-label in NDMs [24,25]. Despite not selective for Na_v_1.4 channels, mexiletine, a class IB antiarrhythmic drug, represents the first line therapy for myotonic syndromes. The drug inhibits the typical myotonic discharges of action potentials favoring muscle relaxation by blocking Na_v_1.4 sodium channels in a use-dependent manner [26,27]. Due to the evidence of effectiveness in the treatment of NDMs, mexiletine received the orphan drug designation in 2018 by the European Medicines Agency [28]. Two independent, randomized, placebo-controlled trials have shown that mexiletine is effective in reducing weakness, tiredness, muscle stiffness and pain [29,30,31,32]. In addition, the efficacy and safety of mexiletine was also recently confirmed in a long-term study conducted in a single center [33]. The drug is also able to improve the stiffness and transitory muscle weakness associated with recessive myotonia congenita due to chloride channel ClC-1 mutations [34]. Nevertheless, due to contraindications, side effects (gastrointestinal discomfort, dizziness, tremor, cardiac problems) or lack of response, several myotonic patients obtain little or no benefits from mexiletine [31,35]. Thus, other sodium channel blockers are offered, as alternative therapeutic option, to myotonic patients and selective Na_v_1.4 compounds would be highly desirable. In sodium channel myotonias, insufficient response to mexiletine may also arise from a reduced affinity of the mutated channel to the drug, and pharmacogenetics studies have suggested that other sodium channel blockers may instead be beneficial in such cases [20,36]. Flecainide and propafenone, class 1C anti-arrhythmic drugs, were proved useful in mexiletine-unresponsive patients carrying specific *SCN4A* mutations, suggesting the possibility of defining a mutation-based pharmacological strategy [37]. Indeed, several mutations located near the fast inactivation gate of the channel and shifting the voltage-dependent fast inactivation towards positive potentials resulted less sensitive to mexiletine but responded well to flecainide [38,39]. An improvement in clinical symptoms and cold-induced electromyographic findings have been reported with propafenone, in a single case of paramyotonia congenita [25]. Despite positive results, because of the existence of a possible pathogenetic linkage between skeletal muscle and cardiac sodium channelopathies, caution must be taken when administering antiarrhythmic drugs to myotonic patients. Indeed, in a recent study, Brugada-associated symptoms were observed after the administration of flecainide, initiated to alleviate a massive myotonic phenomenon [40]. Another class IC anti-arrhythmic drug, tocainide, has been also used in myotonia [41], but it was associated with an elevated incidence of serious adverse events and therefore discontinued in many countries because of potentially fatal agranulocytosis [42].

Other non-antiarrhythmic sodium channel blockers approved for different therapeutic indications provided satisfactory results in non-dystrophic myotonias even though their efficacy is often limited to case reports or small cohorts. Lamotrigine, an antiepileptic drug, has been proposed as a first line treatment for myotonia, in one study of treatment-naive patients and in patients who are intolerant to mexiletine, based on the valuable risk-benefit profile, high availability and low cost [43]. Lamotrigine has been shown to be beneficial in one randomized clinical trial (RCT), and it is now considered the second-choice treatment in several countries [44]. Similarly, ranolazine, a drug used to treat angina known to enhance the slow inactivation of sodium channels, has been proposed as a possible alternative to mexiletine in myotonia congenita non-responders [45,46]. More recently, in an open-label trial of ten patients with paramyotonia congenita, this drug has shown additional significant effect on weakness and pain [47]. The efficacy of non-antiarrhythmic sodium channel blockers such as lacosamide, ranolazine, and buprenorphine was also explored in two patients with paramyotonia congenita [48]. Furthermore, buprenorphine, a drug used to treat severe pain with sodium channel blocking activity, showed promising results for the treatment of exercise-induced paralysis and cold intolerance [48]. Recently, cannabidiol and tetrahydrocannabinol have been tested in a small cohort of patient with drug-resistant myotonia and myalgia in which they showed a beneficial antimyotonic effect achieved through Na_v_1.4 block [49]. Further in vitro and in silico studies supported the therapeutic potential of cannabidiol in myotonia and possibly, to a lesser extent, in periodic paralysis [50]. Other sodium channel blockers may be offered to myotonic patients to treat muscle stiffness and pain, including carbamazepine and phenytoin, whereas quinidine and procainamide are no longer recommended because of adverse effects on cardiac conduction [51]. There are also several other agents with reports of antimyotonic properties mostly demonstrated in small studies or case reports of myotonic dystrophy patients [52,53]. Further studies on a larger number of patients, under carefully controlled conditions, should be considered to address the effectiveness and long-term safety of these therapeutic options.

Other sodium channel blockers are still at a pre-clinical stage of research. Riluzole and lubeluzole, two benzothiazolamines with known voltage-gated sodium channel blocking activity, are under investigation as possible alternatives to mexiletine [39]. Riluzole is currently indicated for amyotrophic lateral sclerosis; although its clinical benefit is still under clarification, it has a good safety profile and neuroprotective effect mediated via inhibition of persistent sodium current [54] Following the antimyotonic activity shown by riluzole in a pharmacologically-induced rat model of myotonia, a human pilot study is going to be launched for NDMs [55]. Safinamide is an add-on therapy to levodopa for Parkinson’s disease. In addition to MAOB inhibition, safinamide inhibits neuronal sodium channels, conferring anticonvulsant activity in models of epilepsy [56,57]. Recently, safinamide has been shown to act as a potent voltage- and frequency-dependent blocker of skeletal muscle sodium channels in in vitro cell models of myotonia. This drug showed antimyotonic activity in a pharmacologically-induced rat model of myotonia [58], supporting the assessment of its antimyotonic potential in humans. Another MAOB inhibitor, rufinamide, was able to block sodium channels in vitro. Its antimyotonic efficacy has been claimed but has not been formally verified in patients [59].

Interestingly, part of the drug discovery process concerning Nav channel blockers, belongs to private drug companies, and results about structure–activity relationship are not available. Then the limited academic research aimed at designing new active molecules, has to rely on the structures of already available Nav channel blockers, as well as on the structure and biophysics of the channels that also strongly contribute to drug effects.

## 3. State-Dependent Block of Sodium Channels: Molecular Basis and Pre-Clinical Drug Studies

As anticipated before, sodium channels are targets for a great variety of drugs, which belong to different chemical classes and include LAs, antiseizure and antiarrhythmic drugs [60]. These small molecules function as blockers of the ion-conducting pore with limited selectivity for the Nav channel isoforms. To maximize the efficacy of treatments while minimizing side effects, it would be fundamental to design compounds able to selectively target the different sodium channel subtypes. As such, structure-based drug design requires a profound knowledge of the molecular determinants for channel binding, mechanism of action, and drug binding sites on the sodium channel protein.

**Figure 1:**
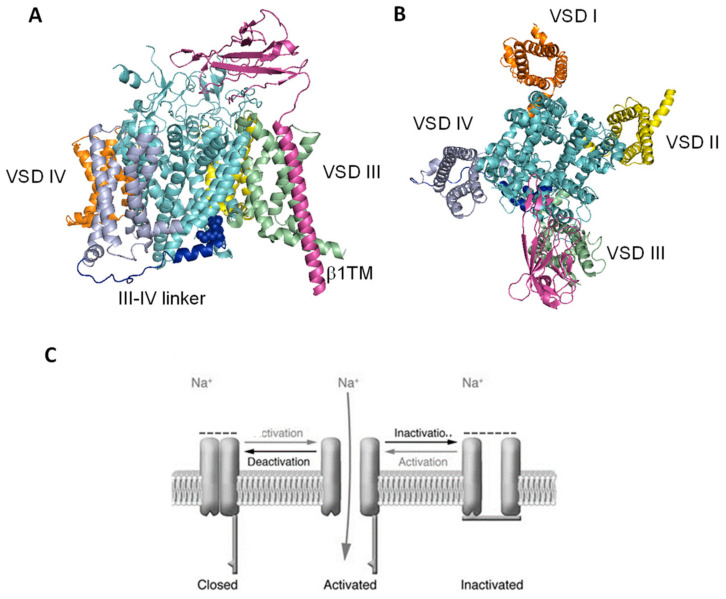
Structure of the human Na_v_1.4-b1 complex. (**A**) Side and (**B**) top view of the human Na_v_1.4-b1 complex (pdb: 6AGF; [64]). The VSDs are colored yellow, orange, green and violet. The S5–S6 helices are colored cyan. The IFM motif is shown as blue spheres, and the III-IV linker is colored blue. (**C**) Schematic representation of an NaVCh undergoing the major gating transitions.

Advances in the structural biology of Nav proteins using X-ray crystallography and cryo-electron microscopy (cryoEM) have provided over the years insight into the molecular basis for their function and pharmacology [61,62,63]. The recent cryoEM structure of the human Na_v_1.4-β1 complex and Nav1.5 [64,65], together with the bacterial Nav structures available, with mutagenesis and computer modeling studies, contributed to clarifying drug binding sites and drug-receptor interactions, thus encouraging drug discovery for Nav channelopathies. Nav1 channel consists of a pore-forming alpha-subunit composed of four homologous domains (DI–IV), each comprising six transmembrane segments (S1–S6), with S1–S4 forming the voltage sensor domain (VS), and S5 and S6 forming the pore module (PM). During depolarization, the VS of the four domains quickly moves the sliding helix S4 outward to activate the channel, and this voltage-dependent conformational change is transmitted to the pore domain through the S4–S5 linker. Fast inactivation, occurs in sodium channels within a few milliseconds of their opening. This process is promoted by the interaction of an isoleucine–phenylalanine–methionine (IFM) motif, located in the DIII-DIV linker, with amino acid residues in the S4–S5 linkers in DIII and DIV and in the intracellular ends of the S5 and S6 segments of DIV (Figure 1) [64,66,67].

The initial molecular mapping of the receptor site, in the rat skeletal muscle Na_v_1.4 channel, for LAs, antiepileptic and antiarrhythmic drugs by site-directed mutagenesis revealed key amino acids in the pore lining S6 segment of domains I, III and IV, consistent with a model where these drugs enter and block the pore [2,67,68,69,70,71,72]. Most LAs and antiarrhythmics are flexible molecules that contain a protonatable amino group at one end, an aromatic moiety at the opposite end, and polar groups in the middle. The main interaction of these compounds involves an aromatic amino acid, Phe1579 lining the pore on DIV-S6 of the Na_v_1.4 channel, but several other residues are likely to contribute to the binding site. It has been proposed that the aromatic part of drugs interacts mainly with the side chain of the phenylalanine residue, while the charged amine group occupies the Na^+^ binding sites in the permeation pathway; in the case of neutral drugs instead, an interaction may occur with the Na^+^ ion trapped in the permeation pathway [73].

The mechanism of blockage of the Nav1 channel is rather complex, and it is generally accepted that sodium channel blockers have different affinities to the different conformational and functional states of the channel (resting, open and inactivated) (state-dependent block). As the binding site is in the pore, the affinity of the drug changes accordingly, depending on the membrane potential and the frequency of action potential generation. The closed (resting) state, which prevails at low-frequency stimulations, is the lowest affinity one (tonic block, TB). Membrane depolarization and an increase in the stimulation frequency favor the interaction of the drug at the binding site, thus enhancing the blocking effect. Thus, the affinity of the drug to the open state of the channel is bigger, whereas the affinity to the inactivated states, which prevails at frequent stimulations, is maximal (use-dependent or frequency-dependent block, UDB). This state-dependent action is essential to allow drugs to preferentially block sodium channels in depolarized, rapidly firing cells as occurring in pain, epilepsy, cardiac arrhythmia, and myotonia without blocking normal action potential generation in unaffected tissues [2,73].

In the search for therapeutically relevant drugs, drug tonic and use-dependent block has been traditionally assessed from sodium current (I_Na_) recordings from adult single frog skeletal muscles fibers by vaseline gap voltage-clamp recording based on methods described by Hille and Campbell [74,75] and later from heterologously expressed Na_v_1.4 channels [76,77]. Different voltage protocols allow the careful assessment of voltage and frequency-related drug-effect and calculation of relative affinities that, in turn, are useful to score compounds based on therapeutically relevant features. The use of mutant sodium channels bearing patients mutations can also help to identify state-dependent channel blockers able to correct specific biophysical defects. In this case, personalized medicine approaches can be pursued [78].

In vivo pre-clinical models can further validate drug action in a more reliable pathology setting. Both the spontaneous mouse model of myotonia, the adr/adr mouse [26,79], and the pharmacologically-induced rat model of myotonia are predictive pre-clinical models used to assess the antimyotonic potential of novel compounds [27,58,76]. The adr/adr mouse presents severe myotonia due to a missense mutation in the skeletal muscle chloride channel gene *CLCN1*. As a result, the macroscopic chloride conductance (gCl), a parameter that guarantees the electrical stability of sarcolemma at rest, is dramatically reduced, leading to pathological hyperexcitability. Then, in myotonic adr/adr mice, skeletal muscles spontaneously generate trains of action potentials triggering involuntary spams, a clinical phenotype very similar to that of Na^+^ channel myotonia [80,81]. In the pharmacologically-induced myotonic rat model, myotonia is generated in adult rats by a single intraperitoneal injection of anthracene-9-carboxylic acid, a known blocker of skeletal muscle ClC-1 channels. This pharmacological maneuver creates a myotonic state similar to chloride channel myotonia [82,83]. The time of righting reflex (TRR), which is the time needed by the animal to right itself on the four legs from its back, is a predictive parameter to monitor an antimyotonic effect at various intervals after drug administration [27,58].

## 4. Structure-Based Drug Design for Na_v_1.4 Blockers with Antimyotonic Activity

Despite the availability of mexiletine and other sodium channel blockers for the treatment of myotonia, pharmacological research is devoted to developing new molecules with high affinity and possibly selective for skeletal muscle Na_v_1.4 channels, with better safety and efficacy profiles, to treat populations with special healthcare needs such as non-responders and patients with heart diseases. In addition, the altered expression of Na_v_1.4 sodium channels as a secondary defect in other neuromuscular disorders [23] suggests the possibility to employ selective blockers with additional peculiar properties in other pathological conditions.

Our group has been committed for many years to characterize the molecular determinants of sodium channel blocking drugs and the interaction between these molecules and skeletal muscle Na_v_1.4 sodium channel [84] with the final goal to develop more potent use-dependent molecules with antimyotonic potential (and thus improved benefit-risk profile). In the last 30 years, starting from mexiletine (Mex) and tocainide (Toc), we performed a large screening of more than 60 newly synthesized analogues of the respective parental drugs, finally drawing the molecular requisites for this class of drugs. Forty-five years ago, Hille [85] proposed two distinct access pathways for LAs to the central binding site, lately confirmed: the hydrophobic pathway through the phospholipidic membrane phase and the hydrophilic pathway through the intracellular gate [2]. This means that the physicochemical properties and specific chemical substituents nearby the pharmacophore groups (the aromatic ring, the chiral center, the amino-terminal group) strongly influence state-dependent drug block. For these drugs, potency is, therefore, the result of two main processes: access to the receptor site located in the channel pore and binding. Lipophilicity can contribute to both aspects, first by facilitating simple drug diffusion through the membrane and then by increasing drug binding to hydrophobic pockets in the channel protein (tonic block, TB) [86,87]. Modifications increasing lipophilicity (logP) or hydrophobic interactions improve drug potency. On the other hand, use-dependent block (UDB) is mostly due to structural modifications that influence the basicity (pKa) of the amino-terminal group. Mexiletine and tocainide, like many antiarrhythmic and LAs drugs, are weak bases that exist in equilibrium between both neutral and charged forms at physiological pH. An increase in the pKa resulting from chemical substitutions will enhance the charged molecule proportion favoring channel block at the intracellular binding site.

**Figure 2:**
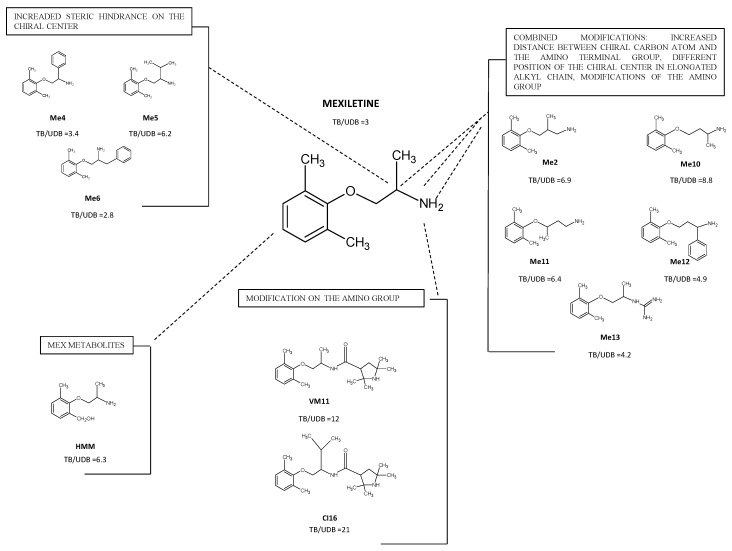
Chemical structure of Mexiletine and its newly synthesized analogs. The boxes show the most interesting compounds with the ratio between IC_50_ values during tonic block (TB) and use-dependent block at the frequency of 10 hertz (UDB) (IC_50_ TB/IC_50_ UDB 10-Hz). The ratio is shown to allow an easier comparison of the use-dependent behavior of each compound [87].

**Figure 3:**
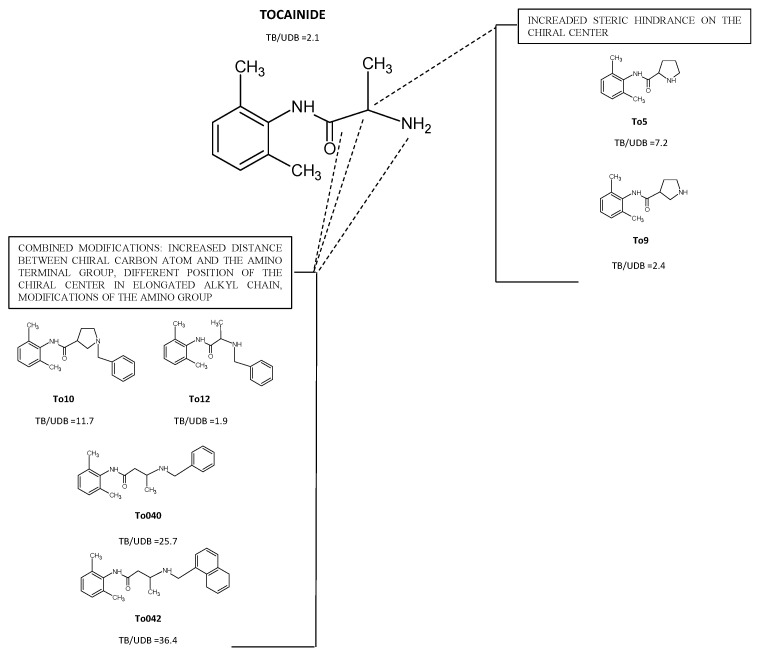
Chemical structure of Tocainide and its newly synthesized analogs. The boxes show the most interesting compounds with the ratio between IC_50_ values during tonic block (TB) and use-dependent block at the frequency of 10 hertz (UDB) (IC_50_ TB/IC_50_ UDB 10-Hz). The ratio is shown to allow an easier comparison of the use-dependent behavior of each compound [89,90].

An overview of the most representative mexiletine and tocainide derivatives studied in our lab is shown in Figure 2 and Figure 3 [88,89]. The new analogs, which have been given arbitrary abbreviated nomenclature, have the following modifications at the pharmacophores groups that conferred improved antimyotonic potential:

1. Increased steric hindrance on the stereogenic center;
2. Increased distance between the chiral carbon atom and the amino-terminal group, different position of the chiral center in the elongated alkyl chain and modifications of the amino group;
3. Introduction of tetramethyl—pirroline moiety of the amino group of Mex.

A summary of the most important compounds is provided in the following paragraphs, together with hints at the role of mexiletine metabolites.

### 4.1. Derivatives with Increased Steric Hindrance on the Stereogenic Center

A clear correlation between the amount of both voltage- and use-dependent block and the lipophilicity of the above substituents has been shown by the replacement of the methyl group of mexiletine with apolar ones, such as a phenyl (Me4), an isopropyl (Me5) or a benzyl (Me6), moiety. This leads to a 3- to 10-fold increase in potency (Figure 2) [87]. Having observed similar results with tocainide analogs, the idea of a common binding site and that the stereogenic center strongly influences the position of the drug molecule at the receptor site was confirmed [26]. According to what is generally known [90,91], tocainide was less potent than mexiletine in animal models of myotonia. In addition, compared to the lead compound, at the dose of 2.5 mg/kg, mexiletine analogues with either an aromatic (Me4 and Me6) or a branched (Me5) group on the chiral cent produced a maximal reduction of TRR ranging between 40 to 55% [27].

Constraining the stereogenic center of tocainide in a rigid proline-like cycle, as in the α proline-like derivative To5, represents an important structural improvement to gain potency. Compared to tocainide, To5, was 5 and 21-fold more potent in producing tonic and use-dependent block of Na^+^ currents, respectively, showing greater lipophilicity due to the proline cycle and greater basicity (a higher pKa value) due to the presence of a secondary amine group (Figure 3) [79].

Although stereoselectivity plays a critical role in the pharmacological activities of molecules, and the chiral carbon atom of both mexiletine and tocainide is an important pharmacophoric part, the stereoselectivity profile of the compounds tested was modest, and this aspect was not investigated further [92,93].

### 4.2. Derivatives with Increased Distance between the Chiral Carbon Atom and the Amino Terminal Group and/or, Modifications of the Amino Group

Potent use-dependent blockers derived from the combination of key modifications, such as the presence of a lipophilic group on the pharmacophore amino-terminal one with the elongation of the alkyl chain, were corroborated by a 3D-QSAR study [94]. Lengthening of the amino-alkyl chain in mexiletine to increase the distance between the chiral carbon atom and the amino-terminal group (Me2) selectively decreased tonic block, thus enhancing use-dependent behavior by a factor of 3 compared to the parent compound [26]. As Me4 and Me2 represent two lead molecules for both tonic and use-dependent behavior, respectively, we demonstrated that the position of the stereogenic center between the main pharmacophore groups may play a role. Compared to Me2, the presence of a chiral center near the amino-terminal group (Me10) or near the aromatic aryloxy ring (Me11) increased potency for producing a TB of I_Na_. Both homologs, Me10 and Me11, showed a strong UDB behavior, suggesting that the activity of Mex-like sodium channel blockers is strongly modulated by the part of the molecule near the asymmetric carbon atom [95]. On the other hand, compared to Me4, any remarkable change in the potency of the drug for TB of I_Na_ has been caused by the combined presence of the phenyl ring and the long chain (Me12). Notably, Me12 showed a higher use-dependent behavior than Me4, which is consistent with the increase in basicity expected from lengthening the alkyl chain [95]. Replacing the amino group of Mex with a more basic guanidine, as in Me13, increased potency in both tonic and phasic block versus mexiletine, positioning itself in the same rank order of use-dependence as Me2 (Figure 2).

Regarding tocainide analogues, the replacement of the amino group with the α- with a β-proline cycle (To5 and To9, respectively) did not significantly change the drug potency and produced a reduction in the use-dependent behavior, probably in relation to less favorable physicochemical properties. Instead, all benzylated derivatives were noticeably more potent than the related unbenzylated parental compounds, with an improved pharmacological profile shown by To10 in terms of potency for use-dependent block and possibly selectivity [76,95,96,97]. Interestingly, the combination of the presence of a lipophilic group on the pharmacophore amino-terminal one, with the elongation of the alkyl chain in the tocainide backbone (To040) strongly increased the potency and the use-dependency of the resulting derivative that was about 70 and even 800 fold more potent than tocainide, respectively, confirming a better interaction with the binding site [97,98,99].

**Figure 4:**
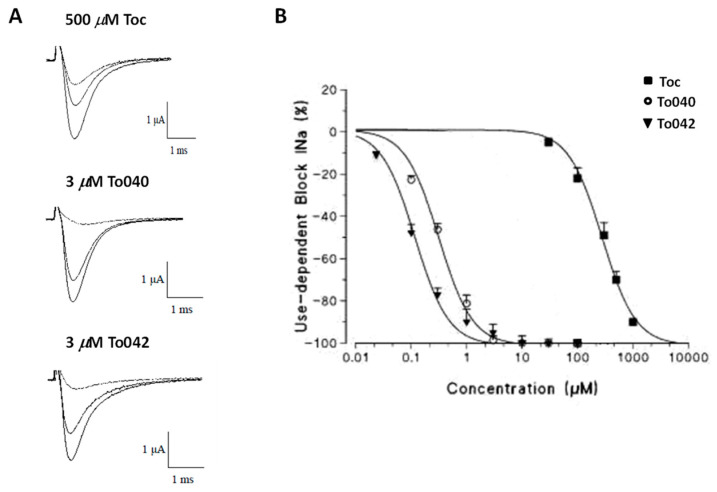
Tonic and use-dependent block exerted by Toc analogues (To040 and To042). (**A**) Traces of sodium current transients recorded in the absence and presence of the test compounds 500 μM Toc, 3 μM To040 and 3 μM To042. (**B**) Concentration-response curves for 10-Hz use-dependent block of sodium currents obtained with Toc, To40 and To42 [96].

The enlargement of the lipophilic substituent as with the naphthyl group at the amino-terminal, in combination with the optimal alkyl chain length (To042), caused an extraordinary increase in the potency, up to hundred and two thousand-fold compared to tocainide for tonic and use-dependent block, respectively. Interestingly, To042 showed the strongest use-dependent behavior among the exploratory compounds tested up to now [76,97,99]. To042 was 100 times more potent for treating muscle stiffness in vivo in a previously validated rat model of myotonia (Figure 4). In this context, To042 appears as a promising candidate to help increase therapeutic options in myotonia and may be of interest for other membrane excitability diseases, such as epilepsy and chronic pain, deserving attention for further pre-clinical studies [27,97]. Recently, a structure–activity relation (SAR) study on To042 has been performed, returning derivatives with favorable pharmacokinetics profiles and enhanced use-dependent behavior [77] (Figure 3 and Figure 4).

### 4.3. Derivatives with Tetramethyl—Pyrroline Moiety of the Amino Group of Mex

**Figure 5:**
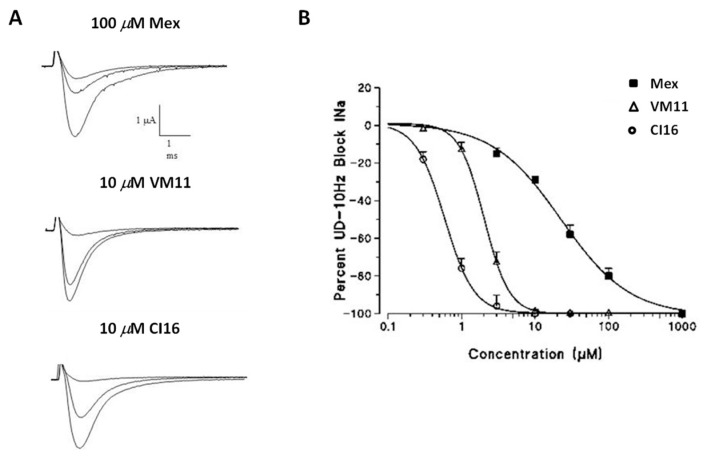
Tonic and use-dependent block exerted by Mex analogues (VM11 and CI16). (**A**) Traces of sodium current transients recorded in the absence and presence of the test compounds 100 μM Mex, 10 μM VM11 and 10 μM CI16. (**B**) Concentration-response curves for 10-Hz use-dependent block of sodium currents obtained with Mex and its tetramethyl-pyrroline derivatives [102].

It has been shown that the pyrroline derivative of Mex and its in vivo nitroxide metabolite show protection against reperfusion injury, probably due to a combination of antioxidant and membrane stabilizing mechanisms [100,101]. Based on these findings, the introduction of a tetramethyl-pyrroline moiety on the amino group of Mex (as in VM11) and of its potent derivative Me5 (as in CI16) improved both the tonic and use-dependent block of Na_v_1.4 channels [102]. Interestingly, CI16 was the strongest use-dependent Mex-like compound so far. The experimental data fitted with the molecular-modeling simulation based on the previously proposed interaction of main pharmacophores with the Na_v_1.4 binding-site (Figure 2 and Figure 5). In addition, in a recent study, CI16 and VM11 were compared to Mex and its isopropyl derivative (Me5) for the ability to protect C2C12-cells from H_2_O_2_-cytotoxicity in the concentration range effective on Na_v_1.4. CI16 and VM11 demonstrated a remarkable cyto-protection at concentrations effective for use-dependent block of Na_v_1.4; instead, Mex and Me5 showed a moderate cyto-protective effect in the presence of H_2_O_2_. This effect was similar to that of selected antioxidant drugs that exerted protective effects in pre-clinical models of progressive myopathies such as muscular dystrophies [103,104].

Thus, the tetramethyl-pyrroline compounds have shown an increased therapeutic profile as sodium channel blockers and an interesting cyto-protective activity. This novel pharmacological profile may expand the therapeutic potential of these drugs from channelopathies to myopathies in which alteration of excitation-contraction coupling is paralleled by oxidative stress, i.e., muscular dystrophies [77,105].

### 4.4. Pharmacological Role of Active Metabolites of Mexiletine

It is known that pharmacologically active metabolites produced during hepatic biotransformation can influence the therapeutic profiles of several antiarrhythmic drugs [106]. Regarding mexiletine metabolites, hydroxy-methyl-mexiletine (HMM; Figure 2) showed marked use-dependent behavior [92] compared to mexiletine and the other phase I metabolites. As hypothesized by other experiments, the presence of substituents on the aromatic ring may modify the orientation of the entire molecule within the binding site [106]. As expected, the glucuronidation resulted in almost a complete abolition of the pharmacological activity of mexiletine [92]. Taken together, these findings indicate that some phase I metabolites, although less potent than the parent compound, behaved as use-dependent and inactivated channel blockers and might partially contribute to the therapeutic efficacy and/or to the long duration of action of mexiletine [92]. Furthermore, the use of prodrugs of mexiletine and its active metabolites has been suggested for the treatment of neuropathic pain and arrhythmias.

## 5. Conclusions and Perspectives

Voltage-gated sodium channels are very interesting as targets for new drugs since they affect a large number of physiological processes and play a role in many diseases. Abnormal functioning of sodium channel subtypes triggers inherited rare channelopathies and acquired disorders, such as non-dystrophic myotonias, characterized by increased sarcolemma excitability and delayed relaxation after muscle contraction [8,17].

Given the rarity of myotonic disorders and the difficulty in conducting clinical trials, the ability to select the best candidates to put forward for such a limited resource is imperative [35]. In this context, our SAR studies have provided key information about the structural requirements of pharmacophores and then a concrete contribution to the development of new lead molecules with enhanced potency, use-dependence and additional cyto-protective profile. In summary, we obtained a chemically optimized tocainide derivative, To042, exerting a very potent and use-dependent block of Na_v_1.4 channels [99]. The main step is now to assess the selectivity of the best candidate(s) toward the other Nav1.x isoforms in order to predict muscle vs. other tissue actions. This would be a crucial step for enhancing the therapeutic interest of the new compounds. Our SAR study is mainly based on a ligand-based approach. To date, the availability of the 3D structure of some human sodium channel isoforms combined with advances in in silico techniques would allow the pursuit of a structure-based approach and to screen libraries of commercial and investigational drugs [107]. Such a combined approach will implement Nav1 channels drug discovery towards more selective drugs as antimyotonic or rather disclose the potential ability of best candidates to bind and modulate other Nav channels still orphan of potent blockers. In addition, in silico screening based on the structural information obtained so far can help the repurposing of already commercially available drugs in orphan disorders in which the modulation of Nav channels is essential. In addition, our studies returned two compounds, CI16 and VM11, with an additional antioxidant activity that could be explored in skeletal muscle degenerative diseases in which both alteration of voltage-gated sodium channels and oxidative stress occur. Furthermore, in view of activity toward other Nav1 channel subtypes, the peculiar pharmacological profile renders these molecules interesting candidates in other orphan diseases, such as several neurodegenerative and epileptic disorders, which would benefit from Nav1 channel block and antioxidant effect [108,109,110]. In this general frame, the physicochemical properties of the best candidates also deserve attention; in fact, a compound optimization strategy can be carried out to assess and improve the pharmacokinetic profile so as to have a muscle-specific delivery or, rather, the ability to cross brain-blood barrier.

## Data Availability

No new data were created or analyzed in this study. Data sharing is not applicable to this article.
